# Carbon-supported ZnO materials for sulfur capturing in supercritical water

**DOI:** 10.1038/s41598-025-98741-2

**Published:** 2025-04-24

**Authors:** Florentina Maxim, Giuseppe Stefan Stoian, Elena Ecaterina Toma, Camelia Nicoleta Borca, Elisabeth Agnes Müller Gubler, Irina Atkinson, Laura Torrent Fabrega, Christian Ludwig, Andrea Testino

**Affiliations:** 1 Institute of Physical Chemistry-Ilie Murgulescu, Laboratory of Chemical Thermodynamics, Splaiul Independentei 202, 060021 Bucharest, Romania; 2 Paul Scherrer Institute, PSI Center for Photon Science, CPS-LSF, PSI, 5232 Villigen, Switzerland; 3 Paul Scherrer Institute, PSI Center for Life Sciences, LNB-EMF, PSI, 5232 Villigen, Switzerland; 4 Institute of Physical Chemistry-Ilie Murgulescu, Laboratory of Oxide Compounds and Materials Science, Splaiul Independentei 202, 060021 Bucharest, Romania; 5 Paul Scherrer Institute, PSI Center for Energy and Environmental Sciences, CEE-LEP-CPM, PSI, 5232 Villigen, Switzerland; 6 Department of Chemistry, Faculty of Sciences, University of Girona, 17003 Girona, Spain; 7 École Polytechnique Fédérale de Lausanne (EPFL), ENAC IIE GR-LUD, 1015 Lausanne, Switzerland; 8 École Polytechnique Fédérale de Lausanne (EPFL), STI-SMX, 1015 Lausanne, Switzerland

**Keywords:** Supercritical water, Sulfur sorption, Carbon-supported zinc oxide, Continuous flow reactor, Materials science, Nanoscale materials, Nanoparticles

## Abstract

Sulfur (S) capturing materials working at supercritical water (SCW) conditions need to be designed and developed to overcome issues related with catalyst poisoning during the hydrothermal gasification of wet biomass, an efficient and sustainable technology for alternative fuels production. Sorbent materials of zinc oxide (ZnO) deposited on porous carbon (C) support were prepared by an innovative continuous flow SCW impregnation method. Their S-adsorption performance was tested under the same supercritical conditions in the presence of sodium hydrosulfide (NaHS), as model inorganic sulfur compound. During sulfidation experiments, ZnS replaced ZnO indicating an efficient chemisorption of S with the formation of the sulfide particles by a pseudomorphic replacement mechanism. The S adsorption capacity of the ZnO/C composites reaches 1.55 mol_S_/mol_Zn_ at relatively low temperature, which is much higher than those of other reported S capturing materials employed in SCW processes. The results reported here confirm that S sorbents can be both generated and used under the continuous flow SCW conditions relevant for technological applications towards the production of hydrogen and methane from biomass wastes and residues.

## Introduction

The supercritical water (SCW) technologies received intensive attention in the last decades as viable solutions for the hydrogen production and for the highly efficient treatment of wastewaters and sludges from a wide variety of industries^1–5^.

Three processes that occur in SCW are the most relevant for energy harvesting and/or recovery from the waste sources: (a) oxidation, (b) gasification and (c) liquefaction. The exothermic oxidation of high concentration organic waste waters at SCW conditions allows the elimination of toxic organics with high efficiency (typically > 99%) (e.g. Ref.^5^) and at the same time the recovery of the renewable energy contained in the feedstock (e.g. Ref.^6^). Oxidation under SCW conditions has the main advantage over classical wet oxidation of decreasing reaction time, as complete destruction of the organic compounds can be achieved in seconds^7,8^. If the goal is to produce a fuel gas with a high energy content, then catalytic SCW gasification (cSCWG) is the preferred conversion route. A main benefit of SCWG technology is that the energy-consuming drying pre-treatment of the organic feedstock is omitted when using water as solvent^9^. SCWG can also be performed without a catalyst, which has been successfully demonstrated with the Verena pilot plant at Karlsruhe Institute of Technology by the team of Boukis et al. (e.g. Ref.^10^). However, the higher temperatures needed may lead to severe clogging and corrosion issues in an industrial plant. The Paul Scherrer Institute has been a scientific leader in cSCWG for the production of biomethane. Catalytic SCWG technology with salt separation can also be used for closing the nutrient cycles. This was demonstrated by integrating an algae production with a cSCWG process^11^. In this process the recovery of inorganic compounds from the hydrothermal process is a promising option for the integrated recycling of the nutrients, i.e. CO_2_ separated from the synthesis gas and salts separated from the hydrothermal brine effluent can be reused to efficiently grow new algae. This option allows biomass production without continuous loss of fertilizers, as the nutrients remain in a closed production cycle. As the production of fertilizers is a key driver for the high ecological footprint in agriculture (gray energy), such technology has the potential to make hors sol biomass production sustainable. Beside the short-term visions of producing bioenergy which is not in competition with the agriculture and food sector, it paves the way for hors sol bioenergy and food production in remote places. How else does Elon Musk want to feed his crew on Mars?

Anyway, before being ready for the Mars mission, some homework needs to be done, as there is still a lack of fundamental understanding of SCW. It is known now that there is an anomalous behavior in the SCW region, with drastic changes in the physical properties of water due to molecular-scale inhomogeneity^12^. Only recently we were able to demonstrate that pseudo-boiling is not only existing in theory, but can be made visible in an experiment^13,14^. A topical book has reported on the status and the open questions related to the process in SCW for environment, energy and nanomaterials applications^15^. The main SCW application under focus in our work is related to the energy harvesting from wet organic streams by SCW gasification processes^1,16^. One of the major issues still to be solved in the field of cSCWG of the biomass to methane or hydrogen is related to the fact that the catalyst is extremely sensitive to poisoning already at sulfur concentrations of a few ppm in the SCW phase^17^.

Under less severe conditions, some materials, such as ZrO_2_, TiO_2_ and Fe_2_O_3_, also show some catalytic activity towards the synthesis of olefins, ketones, and aromatic aldehydes^18^ and, to some extent, a S-resistant performance. This behavior suggests the possibility to use metal oxides (Me_x_O_y_) as S-capturing materials^19^. Several approaches exist to mitigate the catalyst poisoning by S. Adsorption is the most convenient method from the economic viewpoint^20^. The efficiency of the adsorptive desulfurization is influenced by process parameters such as the contact time, the initial sulfur content of the feedstock, and the possibly to easy regenerate the spent adsorbent^21^. For energy savings, the S adsorption has to be done in SCW to avoid the modification of the steam temperature or pressure necessary if using conventional S-capturing processes that means to separate S-sorption from gasification.

High temperature Zn-based sorbents were developed for highly efficient desulfurization of hot coal gas, with strong abilities of absorbing H_2_S and suppressing COS-release^22,23^. In addition, metal oxides such as ZnO, NiO, MgO, CuO, TiO_2_, CeO_2_, MnO_2_, ZrO_2_, were studied as S-capturing materials^24–26^. However, limited information is available for their activity and stability at SCW conditions^27,28^. Very active materials can be produced by tailoring the metal oxide particle size, morphology, and crystalline phase, which define the specific surface area available, the prevalence of specific crystallographic planes, and thus the surface chemical interaction with S. Therefore, the sorption capacity might be greatly affected by the synthesis method of the active Me_x_O_y_^29^. The use of supported Me_x_O_y_ nanoparticles is practical and cost-effective, as it helps to maintain their performance and allows for easier regeneration and reuse^30^. Preparation of supported (nano)particles by impregnation is the usual procedure, followed by drying and eventually activation to obtain the active material. The loading on the support and its dispersion within the support pores depends significantly on the conditions employed during impregnation and drying^31^. By conventional methods, redistribution of active metal oxides can occur during drying, which in turn lowers the sorption capacity. The supercritical water impregnation (SCWI) method has been developed as a simple procedure employing SCW to obtain highly dispersed metal/metal oxide nanoparticles on porous support, without the use of toxic or noxious solvents, and without the need of post-treatments, such as calcining or drying. However, according to our best knowledge, in the literature there are only few reports on the preparation of composite materials by SCWI method in batch reactor^32–39^. Supercritical water impregnation method was first proposed by Otsu and Oshima in 2005, for the deposition of manganese oxide, silver and lead oxide on porous alumina support^32^. In this first work, as well as in the following group’s works^33,34^, they showed that SCWI can be a promising method for the preparation of catalytic materials made of inorganics deposited on porous support. They showed that the crystallinity and the composition of the products can be controlled by temperature and pressure. The impregnation time, as well as the preparation temperature during SCWI, were found to be the main influencing factors for the desulfurization activity of sorbents made of Cu, Mn and Zn oxide particles on activated carbon. These factors behave mainly by changing the micropore volume and surface area of sorbents and the dispersion of metal oxide particles on the support^38^. Moreover, it was suggested that by applying a continuous reactor, enough product amounts can be obtained in a single experimental run^34^. In the case of flow reaction systems, the density of water can be varied with the temperature and pressure during the reaction, like that better control of the products crystallinity and composition could be obtained. A very recent study of our team reports on an innovative continuous flow SCWI approach for the preparation of functional materials made of zinc and copper oxides deposited on activated carbon fibers^40^.

In the present work, we focused on testing the S-capturing capacity of ZnO/C materials in a continuous flow SCW reactor operated at conditions suitable for the catalytic hydrothermal gasification of wet biomass. The samples of this study were characterized by various techniques, including XRD, SEM, TEM, XRF and ICP-OES.

## Results and discussion

### ZnO/C composites used as sorbent materials

**Fig. 1:**
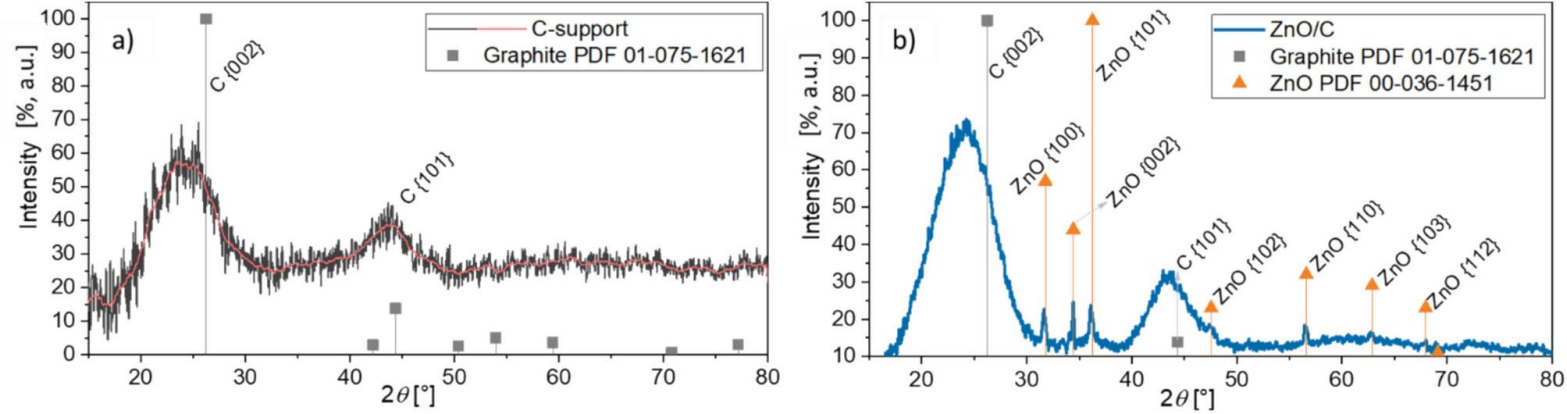
XRD patterns of (**a**) C monolith used as support for impregnation and (**b**) ZnO/C material obtained by supercritical water impregnation method.

Figure 1 presents the XRD patterns of the C monolith (Fig. 1a) used as support, and of the ZnO/C composite (Fig. 1b) obtained by the supercritical water impregnation method at 668 K, 250 bar, 5 mL min^−1^ flow rate and 4 mmol L^−1^ of Zn^2+^ in the precursor solution. These results show that the XRD pattern of the support can be assigned to the graphite structure of C, and that the ZnO/C composite materials, obtained by SCW impregnation consist of hexagonal structured zinc oxide.

**Fig. 2:**
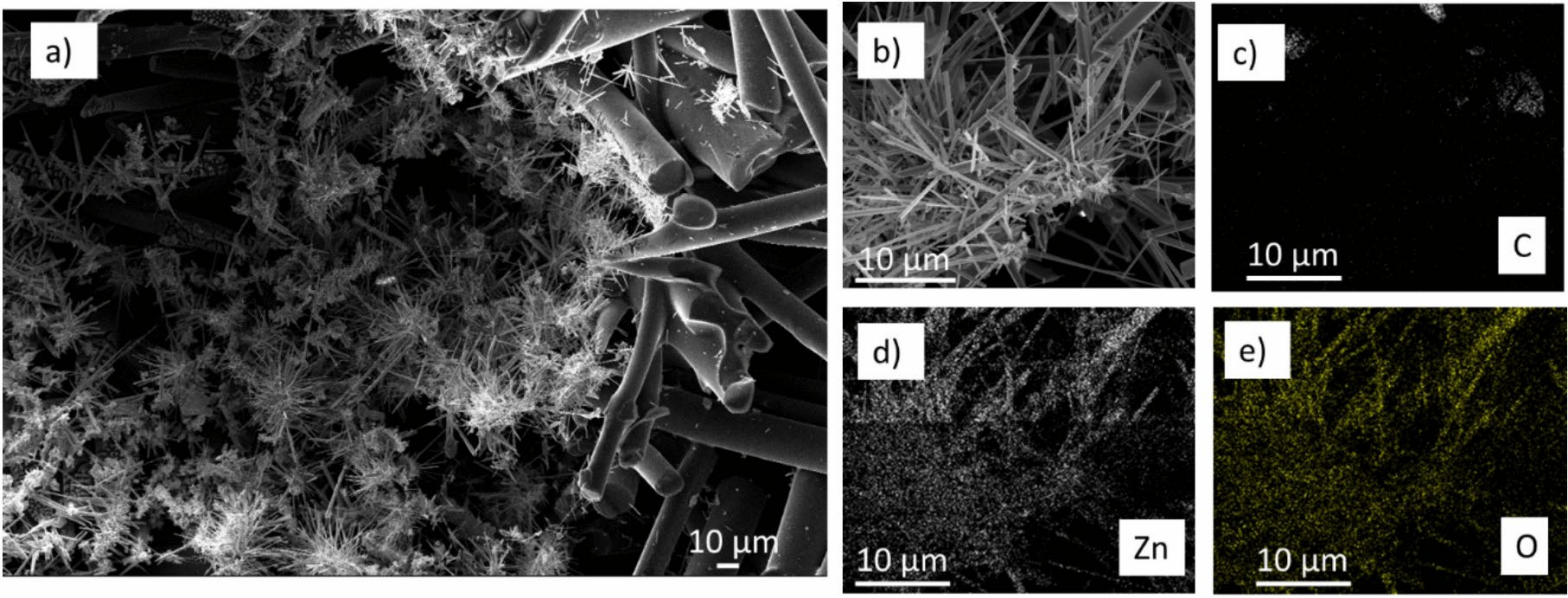
(**a**,**b**) SEM micrographs (increasing order of magnifications) of ZnO/C composites prepared by supercritical water impregnation at 668 K, 250 bar; (**c**–**e**) elemental mapping of C, Zn and O; 1D ZnO needle crystals are formed in the interfibrillar voids of C support.

**Fig. 3:**
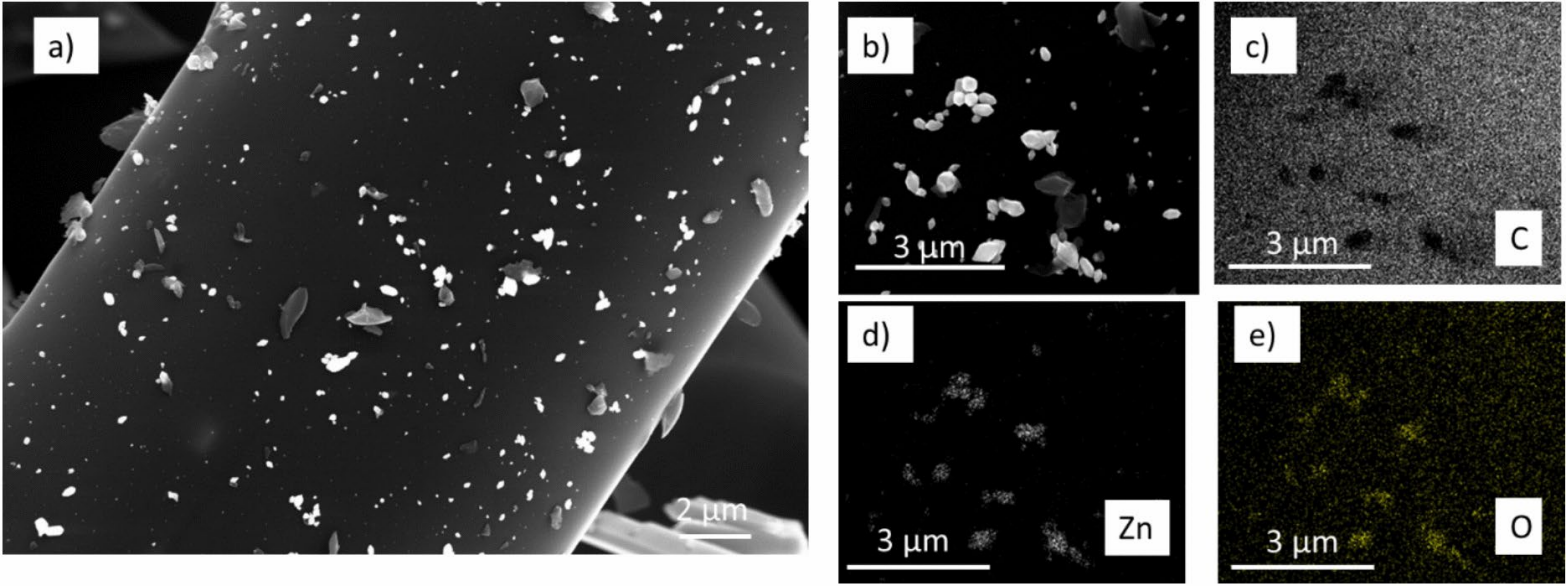
(**a**,**b**) SEM micrographs (increasing order of magnifications) of ZnO/C materials prepared by supercritical water impregnation at 668 K, 250 bar; (**c**–**e**) elemental mapping of C, Zn and O; ZnO particles deposited on the C fiber surface.

In Figs. 2 and 3 the SEM micrographs of ZnO/C samples and their EDS elemental analysis maps are shown. It can be observed that, within the interfibrillar voids of the carbon matrix, ZnO particles with 1D morphology are formed (Fig. 2a,b). In addition, under the same supercritical impregnation conditions, ZnO particles with cuboidal/hexagonal shape are deposited on the C fibers, as shown in Fig. 3a,b. The elemental mapping of C, Zn and O presented in Figs. 2c–e and 3c–e confirm that both type of particles consist of Zn and O elements.

The Zn content in the ZnO/C sample determined via the first XRF method (see “Experimental” section) it was measured to be about 0.2 wt% indicating low loading of the metal oxide on the porous carbon support (Table S1). It is to mention here that, a certain amount of sulfur is detected by XRF to be contained in the C raw material used as support. This amount of sulfur remains even after the impregnation of ZnO (Table S1 and Fig. S1), and it is assumed to be reminiscence of the C support preparation method^41,42^.

**Fig. 4:**
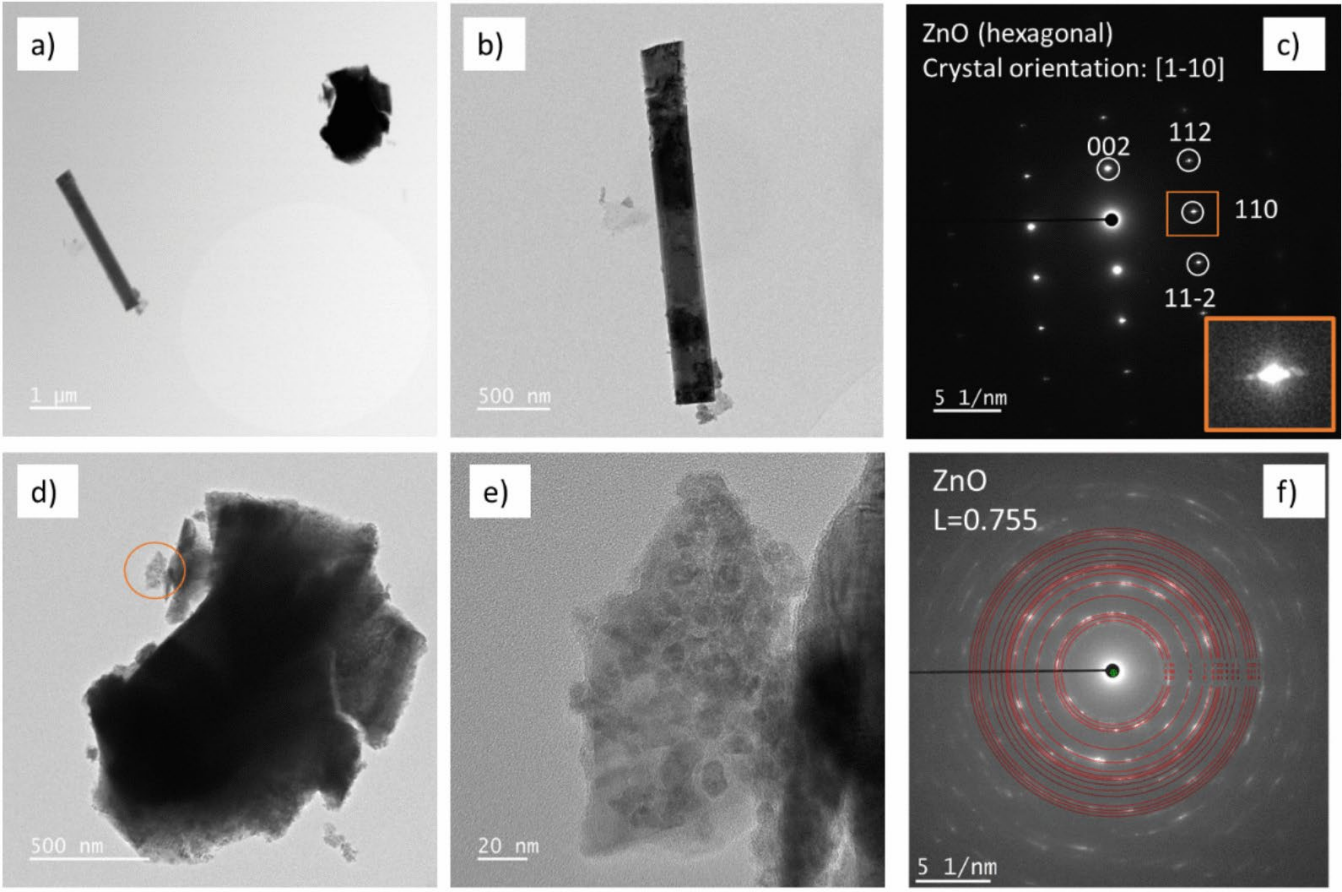
TEM analysis of the ZnO/C materials. (**a**) BF STEM image of the two types of particles; (**b**) and (**d**) higher magnification TEM images of the particles shown in (**a**); (**c**) and (**f**) electron diffraction patterns of the two types of morphologies: rod-like and assembly of small crystalline particles presented in orange circle in (**d**); (**e**) higher magnification of marked zone in (**d**); the inset in (**c**) shows the spot splitting associated to the twining in the ZnO structure; L in (**f**) is the camera length.

Figure 4 presents the analysis of the ZnO/C sample obtained by transmission electron microscopy (TEM). In Fig. 4a the bright field STEM image, show two types of particles morphologies rod-like (1D) and sheet-like (2D). In Fig. 4b,d, TEM images acquired at higher magnifications of these two morphologies are presented. The different contrast in the dark field images (not shown) reflect the different particles thicknesses of the same region of interest and a dissimilar degree of crystallinity. From the diffraction patterns of these regions (Fig. 4c,f) it can be concluded that the rod-like particle consists of single crystal ZnO (Fig. 4c). It is important to mention that, there are some spot splitting recognizable in the diffraction patterns of the rod-like particle (inset in Fig. 4c), which suggests the formation of defect ZnO structure by twining^43^. The formation of such self-assembly ZnO twinning nanostructures were reported to follow a growth model based on the vapor–liquid–solid mechanism^44^. The 2D sheet-like object, however, is composed of very small crystalline particles confirmed by HRTEM image of thin regions of the big particle, in which the small particles can easily be recognized (Fig. 4e). These give rise to the indexed diffraction pattern corresponding to hexagonal ZnO nanoparticles (Fig. 4f).

Composite materials made of ZnO on C-based support were prepared by conventional hydrothermal synthesis, in Teflon-lined autoclave, at temperatures not higher than 150 °C and reaction time up to 24 h. However, the conventional procedures to obtain ZnO/C materials are performed at least in two steps, being needed the pre-deposition of ZnO as a seed layer^45–47^, the use of highly concentrated mineralizer for precipitation and growth^45,48^, and the post treatment, such as calcination^49^ in order to obtain crystalline product.

In the works by Oshima’s group, first reporting on SCWI method development in a batch reactor, it was pointed out that particles can be crystallized onto the support surface, as well as they could formed into the pores of alumina/activated carbon by increasing the reaction time from 1 to 60 min^32–34^. They proved that longer reaction times (impregnation time under SCW conditions) can deliver equivalent samples, in terms of particles dispersion onto activated carbon support, as the samples obtained after pre-immersion in the precursor solution at room temperature for days (up to 1 month)^33^. This result suggests that the immersion step used to deposit, for example, Fe_2_O_3_ particles on activated carbon^35^ or on silica^36^ and CuO on silica^37^ can be skipped. In the continuous flow conditions of our experiments, we set to 1 h the reaction/impregnation time in order to ensure the necessary Zn to C ratio for uniform distribution of deposited particles. Actually, extension of reaction time affects the diffusion steps into the pore, a crucial issue determining the mechanism of particles deposition^34^. Our previous study demonstrated that, during impregnation of ZnO on porous activated C support, in gas-like SCW, two mechanisms of particle deposition are possible, as also reported in^34^ for silver nanoparticles deposited on porous alumina support by SCWI. There are 1D ZnO particles grown in the bulk phase of macropores forming interfibrillar voids into the C support^13^, followed by adsorption to the support surfaces^34,40^. In the second mechanism, the formation at the support surfaces of ZnO nanoparticles is most probably favorized by the presence of micropores within the C fibers^13,40^.

### Supercritical S-capturing capacity

**Fig. 5:**
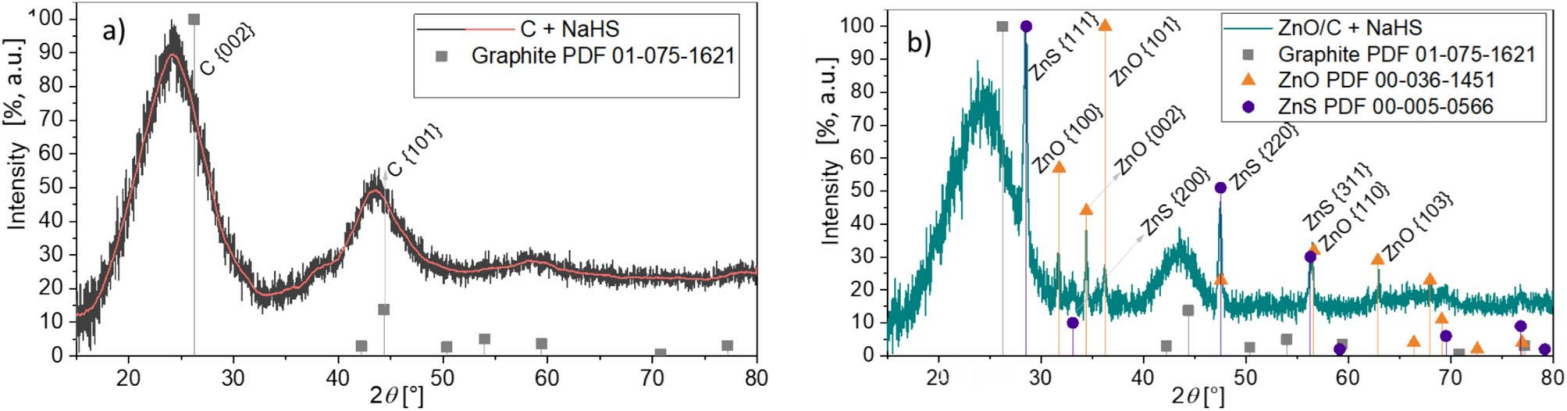
XRD patterns of samples exposed to NaHS (aq) during supercritical water desulfurization experiments in presence of (**a**) C support and (**b**) ZnO/C composite.

In the catalytic SCW gasification of wet biomass to produce gas fuel, the presence of organic/inorganic sulfides contribute to the catalyst poisoning via irreversible adsorption of S^2−^^50^ We choose NaHS as model inorganic sulfide because it contains S^2−^ and HS^-^ species that are relevant for the sulfidation reaction of ZnO, and, in the same time, it is less corrosive than Na_2_S. Figure 5 presents the XRD pattern of the samples collected after desulfurization experiments with only C support material (Fig. 5a) and in the presence of ZnO/C composites (Fig. 5b).

The eventual modifications of the raw C support were not detectable by XRD (Fig. 5a). Like before the desulfurization experiment (Fig. 1a), the pattern can recall a graphitic structure. By contrast, in the case of the desulfurization experiment in presence of the ZnO-containing material, the formation of ZnS structure has been revealed by XRD (Fig. 5b), however, it is to note that not all oxide has been converted to the sulfide.

**Fig. 6:**
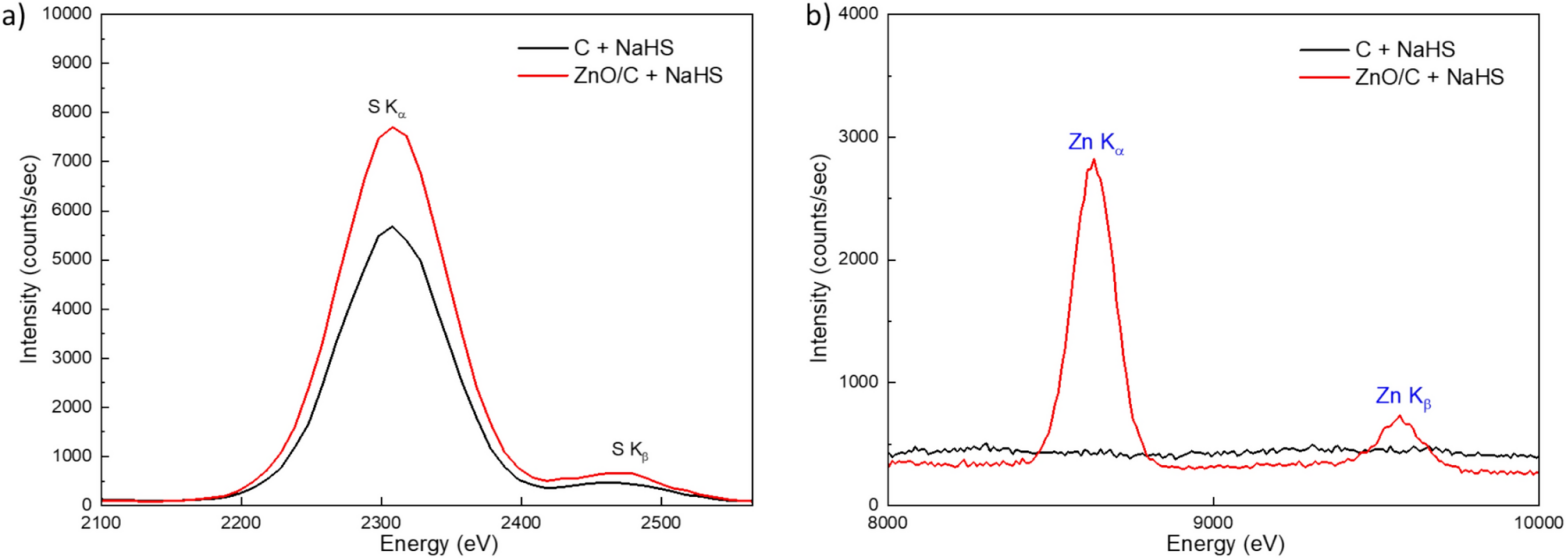
Fluorescence spectrum after desulfurization experiment on ZnO/C + NaHS (red) compared with spectrum of reference sample C + NaHS (black); (**a**) Main fluorescence K_α,β_ lines are identified with S element and (**b**) Main fluorescence K_α,β_ lines are identified with Zn element.

The quantitative analysis of S adsorption on the composite materials obtained by impregnation under SCW conditions was carried out by the second high resolution XRF method and ICP-OES (see “Experimental” section). The fluorescence spectrum for the resulting sample after the desulfurization experiment on ZnO/C is shown in Fig. 6, for the S (Fig. 6a) and Zn (Fig. 6b) regions, compared to the fluorescence spectrum of the reference sample that is the C support exposed to NaHS under the same conditions. From the analysis of the areas of sulfur fluorescence lines (K_α_ + K_β_), calculated with a Gaussian fit (Fig. 6a), it was determined that sulfur adsorption is 2.98 times higher when ZnO/C is used as adsorbent material than the reference C sample. The background for the Zn fluorescence lines in Fig. 6b was not subtracted for either samples, because it is due to surface roughness scattering of the C supports, which is very different when using a 2 mm X-ray beam. The enhanced sulfur adsorption on ZnO/C samples is also confirmed by ICP-OES analysis, which indicates a sulfur concentration of 19,100 mg kg^-1^. It is to mention that, due to some practical limitations, the refence sample (C + NaHS) was not analyzed by ICP-OES. After subtracting the S contained into the sample before adsorption, it was calculated by ICP-OES an adsorption capacity of 1.55 mol_S_/mol_Zn_, a value much higher than the 0.11 mol_S_/mol_Zn_ reported for ZnO/C materials in the only study presenting results of S-sorption in batch SCW reactor working at temperature/pressure conditions similar to our experiments^20^. Expressed in mg S per g sorbent, it was calculated an average adsorption capacity of 7 mg S/g (relative standard deviation of 15%) a value much higher than the 1.1 mg S/g reported in^38^ for ZnO/C sorbents prepared by SCWI for removal of H_2_S from hot gas. For the reactive adsorption of H_2_S at room temperature it is reported a value of around 30 mg S/g for ZnO/C prepared by incipient wetness impregnation with aqueous solutions of Zn(NO_3_)_2_ followed by drying at 100 °C and thermal treatment in N_2_ flow for 3 h at 250 °C^51–53^.

The tests for S adsorption capacity under SCW conditions and in the presence of NaHS aqueous solution, as model inorganic S source, reveal that ZnS is formed (Fig. 5) and S is captured almost three times more than the reference C sample when ZnO/C is used as sorbent material (Fig. 6). This experimental result confirms our predictions, obtained by computational fluid dynamics, that indicated an improved S-capturing on sorbent bed with complex geometries^54^. However, this improved S adsorption capacity determined by experiment, in spite of a low amount of ZnO, it cannot be addressed only to the ZnO/ZnS chemical reaction, considering that ZnO is not quantitatively converted to ZnS (Fig. 5b) and S is contained also in the C + NaHS sample (Fig. 6a). Therefore, we address the deviation from the 1:1 stoichiometry to the S doped into the C matrix. A very interesting result reported in the literature shows that sulfur, when doped into a carbon matrix, can stabilize ~ 1 nm metal nanoclusters (Pt, Ru, Rh, Os, and Ir) at high temperatures up to 700 °C. The authors found that the interfacial metal-sulfur bonding is responsible for the improved adhesion strength between metal nanoclusters and the sulfur-doped carbon support^55^.

**Fig. 7:**
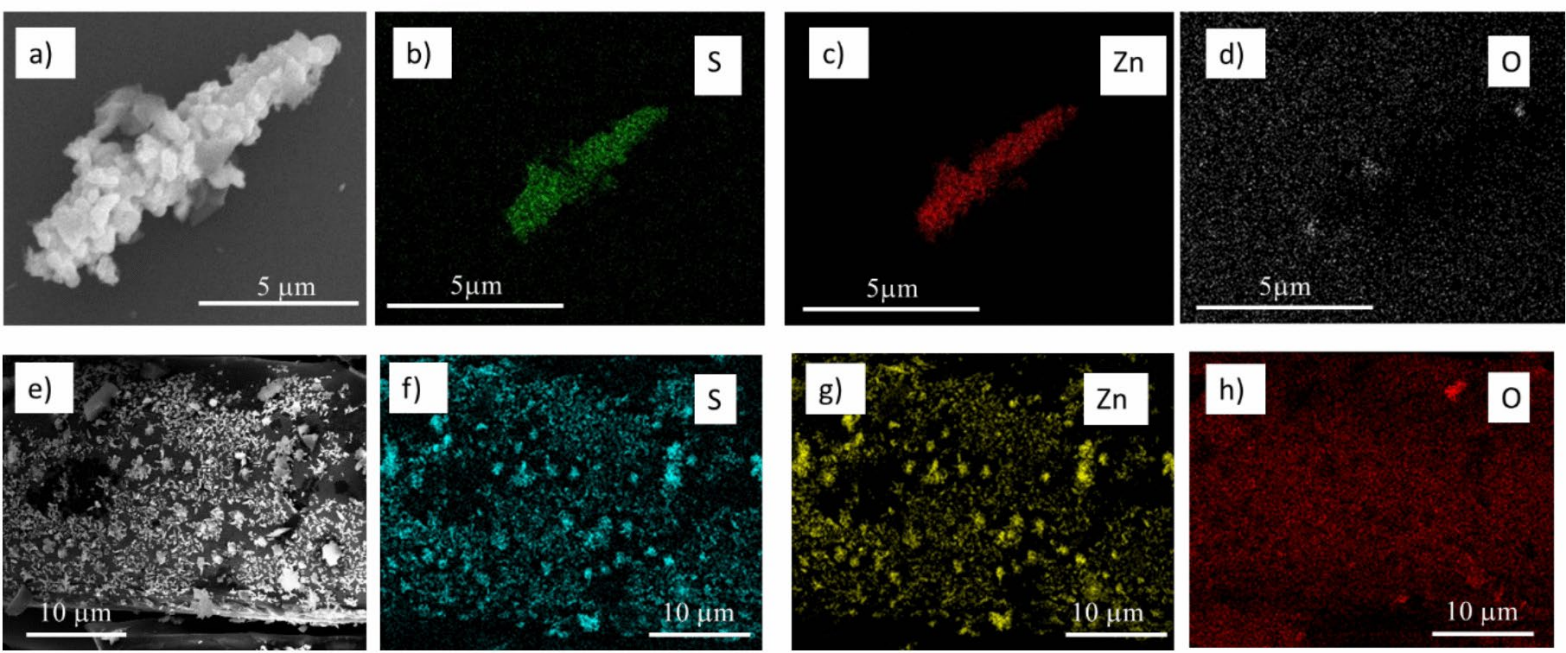
SEM analysis of sorbent samples obtained after desulfurization experiments over ZnO/C; (**a**,**e**) SEM images of elongated polycrystalline particle and particles covering the C fiber; (**b**–**d**) and (**f**–**h**) the above-mentioned regions corresponding elemental maps for S, Zn and O, respectively.

Figure 7 presents the SEM and EDX analysis including the elemental maps for S, Zn and O, of the sorbent samples obtained after exposure to NaHS in supercritical conditions. The SEM image (Fig. 7a) shows an aggregate of particles formed on rod-like skeleton attached to the C fiber. The EDX-maps (Fig. 7b–d) confirm the ZnS composition. In Fig. 7e–h a C fiber section is presented. As expected from Fig. 3a, Zn is found here in the form of particles—also here co-located with S covering the support surface as ZnS particles.

**Fig. 8:**
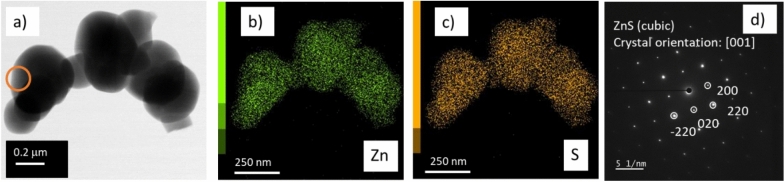
TEM analysis of spent ZnO/C sample after S-adsorption experiment with NaHS (aq) under SCW conditions; (**a**) BF STEM image; (**b**,**c**) elemental maps of Zn and S; (**d**) electron diffraction pattern in the region marked with orange circle in (**a**).

The ZnS particles aggregates were analyzed by TEM and the images are presented in Fig. 8. This analysis confirms that ZnS particles are formed after the SCW exposure of ZnO/C sorbent composite to NaHS aqueous solution.

**Fig. 9:**
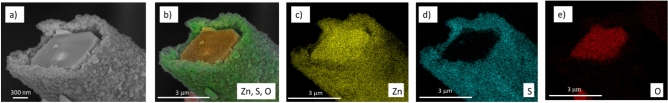
SEM analysis of spent ZnO/C sorbent material after S-capturing test with NaHS (aq) under continuous flow SCW conditions.

Figure 9 presents SEM micrographs together with the elemental analysis obtained by EDX for the rod-like particle formed into the spent ZnO/C sorbent after the desulfurization. This particle probably represents an intermediate stage of conversion, where a core of ZnO is surrounded by a polycrystalline layer of ZnS, with an empty gap in between. This peculiar morphology recalls a pseudomorphic replacement mechanism^56^, where the initial ZnO particle morphology is maintained upon phase transformation. This kind of local chemical transformation, rather known in mineralogy, may compete with a more common dissolution–recrystallization process, which would take place when fast dissolution is allowed. We may speculate that the transformation from ZnO to ZnS, when it takes place under SCW conditions, might be relatively slow due to the extremely low salts solubility in that condition. Therefore, alike crystallization processes in hydrothermal vents, pseudomorphic replacement may take place.

For relatively high dimension 1D particles, the results indicate that pseudomorphic replacement may occur and polycrystalline layer of ZnS on ZnO core is formed (Fig. 9). The gap in between the two materials may be ascribed to the density difference between ZnO and ZnS. The ZnO core might be protected from further transformation due to the diffusion barrier offered by ZnS, preserving the core and providing the residual ZnO reflection in the XRD pattern (Fig. 5b). This finding may suggest that small ZnO supported particles are required to avoid such a core–shell structure, which may limit the chemical reaction responsible for S capturing.

Our findings related to the pseudomorphic replacement under SCW conditions might be an interesting synthesis method to produce pseudomorphic ZnO-based heterostructures, reported to be good candidates for solar cell nanostructures, especially the semiconductor nanowires^57^. The ALD-deposited radial ZnO-core/ZnS-shell nanowire heterostructures exhibits high optical quality^58^. It was found that the properties of such heterostructures are mainly related to the lattice mismatch between the ZnO-core and the pseudomorphic layer of the shell with a critical thickness^59,60^.

SEM and TEM analyses disclose that both types of zinc oxide particles are transformed into zinc sulfide under the continuous flow supercritical conditions of this study (Figs. 7, 8 and 9). Our results indicate that the conversion of the ZnO to ZnS is predominately made through the sulfidation reaction^61^, and its mechanism and kinetics depend on the particles size and shape^62^. In the present study, the relatively small cuboid-shaped, as well as the large one-dimensional ZnO particles are transformed to ZnS by pseudomorphic replacement, the difference being in the rate of replacement which depends on the characteristics of ZnO/ZnS interface^63^. For the environmental sulfidation of ZnO NPs in the presence of aqueous solution of Na_2_S, it has been found that the solubility of partially sulfidized ZnO is controlled by the remaining ZnO core and not quenched by a ZnS shell formed^61^. For larger particles, the ZnO sulfidation by H_2_S was reported to be kinetically controlled by the oxygen diffusion trough the ZnO–ZnS internal interface and by the external interface reaction with water desorption. Void formation at the internal interface leads to a decrease in the internal interface and to a strong slowing down of the reaction rate^62^. Our results are consistent with these findings showing that the voids formed at the ZnO–ZnS interface slows down the reaction rate and unreacted ZnO core remains after the sulfidation on one-dimensional particles (Fig. 9).

## Conclusions

The present study focused on proving the reliability of the method to produce ready-to-use S-sorbent materials functional under continuous flow SCW conditions, similar to those used for the catalytic hydrothermal gasification process of wet biomass. ZnO/C sorbent materials were successfully prepared by the continuous flow impregnation in gas-like SCW, starting from zinc nitrate as Zn source and using neither seed layer nor mineralizer for the ZnO particles growth on porous support made of activated C fibers. Particular features of the C support influence the formation of hexagonal structured ZnO particles with two types of morphology: one-dimensional ZnO particles and cuboid-shaped ZnO particulates.

The S-capturing capacity of as-prepared composite materials was tested under conditions similar to those used for the catalytic hydrothermal gasification process of wet biomass, and in the presence of model inorganic sulfide compound to mimic the S poisoning environment of real feedstocks. The sulfidation experiments revealed that ZnS is formed in place of ZnO by chemical reaction. In case of relatively large ZnO rods-like particle, pseudomorphic replacement may occurs, limiting the complete conversion and highlighting the need to deposit relatively small particles for an efficient desulfurization process. We achieved quite high S-capturing capacity as compared to the reference C sample. This interesting experimental evidence demonstrates that ZnO/C sorbent materials can be generated in situ and offer improved sorption capacity under SCW conditions with respect to other sorbents reported in the literature. This approach demonstrates that ready-to-use sorbent materials can be produced and tested under continuous flow SCW conditions.

## Methods

### Materials

Activated porous carbon (C), provided by Oak Ridge National Laboratory (USA) were used as support. The support samples are monolithic-type with hexagonal graphite structure and constituted by activated carbon fibers with an average diameter in the micrometric scale (10–20 µm) (Fig. S2)^13^. For this study, C material with the following characteristics was used: specific surface of 1100 m^2^ g^−1^, total pore volume of 0.59 cm^3^ g^−1^ for pore radius lower than 180 nm and a water absorption capacity of 165 wt% at standard conditions.

Analytical grade zinc nitrate (Sigma Aldrich) was used as Zn^2+^ precursor. Aqueous solutions of different concentrations ranging from 4 to 16 mmol L^−1^ were prepared by dissolving the corresponding amount of Zn(NO_3_)_2_·6H_2_O in ultrapure, degassed water.

As S source, sodium hydrosulfide (NaHS·xH_2_O, Sigma Aldrich) was used without further purification. Stock aqueous solutions were prepared by dissolving 4.37 g of sodium hydrosulfide in 495.62 g ultrapure, degassed water. Before each sulfidation experiment, fresh NaHS solution was prepared from the stock solution by successive dilutions in order to obtain a pH value of 10. The exact S content in solution was measured via chemical analysis.

### Preparation of ZnO/C sorbent materials by SCW impregnation method

All the experiments of this study were performed using the continuous flow SCW tubular reactor of the NISA (Neutron Imaging Supercritical-water Analysis) equipment, which was detailed in Refs.^13,14^. In short, NISA consists of a vertically oriented tubular reactor of 40 mL volume made of Zircaloy 4, inner diameter of 12 mm, with aluminum block heater and embedded preheater. The fluid (water/aqueous solutions) is fed in upwards the reactor by high-pressure liquid chromatography pumps, and it is pressurized via a back pressure regulator. Temperature sensors and pressure gauge are placed along the line and the conductivity of the inlet and outlet aqueous solutions are measured with conductivity sensors. The working parameters, such as temperature, pressure, mass, flow rate and conductivity are monitored on-line and recorded every 10 s via data acquisition software developed in LabView. The temperature inside the reactor recorded by the inner thermocouple it is the real fluid temperature, and it is considered the reference temperature of the reactions.

The preparation procedure of the ZnO/C materials was detailed in our previous work^40^. Briefly, ≈ 0.2 g of C-monolith cylindrical sample of diameter equal to the internal diameter of the reactor, it was placed on the reactor’ thermocouple protection tube amid small screws, as shown in Fig. S2a. Like this, the solid sample acts as a fixed bed, easy to be inserted and recovered into and from the reactor, respectively. The SCW reactor was filled with water at different flow rates ranging from 2.5 to 7.5 mL min^−1^ and pressurized afterwards at 250 bar. When water inside the reactor reached 668 K at 250 bar, the diluted Zn^2+^ precursor solution was fed in and the impregnation under steady state conditions was performed for 1h. After the impregnation, the reactor was cooled down at 623 K with the Zn^2+^ solution flow, while maintaining the pressure at the supercritical value of 250 bar. It is to mention here that in the present study, we used diluted salts solutions and we monitored the conductivity at both reactor’s inlet and outlet. In Fig. S3a it is shown that the conductivity is constant during the impregnation experiment, indicating no substantial changes in salts solubility. After the fluid inside the reactor reached 623 K, it was further cooled down and depressurized with water flow by natural cooling and gradually releasing the back pressure regulator valve, respectively. When water inside the reactor reached the room temperature, the reactor was emptied by flushing pure N_2_. The sample of ZnO/C was collected afterwards and dried at 333 K overnight.

### Sulfidation experiments

For the sulfidation test, the as prepared sorbent material was loaded into NISA reactor using the same procedure as explained above, and supercritical conditions were reached at 668 K and 250 bar with water flow at 5 mL min^−1^. When supercritical conditions of the gas-like state of water were stabilized, fresh diluted NaHS solution of pH = 10 was fed into the reactor and desulfurization run was performed for 1h. It is to point out here that no solubility data or phase behavior under supercritical conditions are reported in the literature for the salts used in this study, namely Zn(NO_3_)_2_ and NaHS. According to Valyashko’s classification, which is based on a correlation between the salt solubility and its melting temperature, there are two types of salts: type I and type II salts^64^. Moreover, Valyashko’s classification is valid for salts with melting temperature higher than the critical temperature of water^64^. At standard pressure, the melting temperature of Zn(NO_3_)_2_ is around 383 K (110 °C)^65^, while for NaHS is 623 K (350 °C)^66^, therefore the above classification is not applicable. As mentioned above, we used diluted aqueous salt solutions and we recorded the conductivity at both reactor’s inlet and outlet. Right after the scheduled sulfidation time, the system was cooled down and depressurized with water flow. The breakthrough curve was determined by on-line monitoring the effluent conductivity during the sulfidation experiments (Fig. S3b). The spent sorbent material was collected and dried using the same procedure as described in the above subsection.

### Materials characterization techniques

Structural characteristics of the obtained samples were determined by room temperature powder X-ray diffraction (XRD), performed on Rigaku Ultima IV equipment with Cu K-α radiation beam. The signals were collected from 10° to 80° with a step size of 0.02°, and a scan speed of 2° min^−1^. The phases identification was performed using Rigaku’s PDXL software and the ICDD PDF-2 database.

Microstructural and morphological characterization of samples obtained from impregnation and sulfidation experiments were performed on a ZEISS NVision40 FIB/SEM, equipped with ESB, SE2, inlens, QBSE, STEM and EDX (Oxford) detectors. The transmission electron microscopy (TEM) analysis of obtained samples was carried out on a JEOL JEM-ARM200F TEM equipment. This microscope is equipped with a cold FEG electron source, a probe corrector, two EDX detectors (JEOL) and with a variety of STEM detectors (ADF, BF, ABF). The samples were crushed in an agate mortar and dispersed in ethanol. A drop of the resulting suspension was deposited on a Cu holey carbon film grid.

The quantitative analysis of the chemical elements contained in the obtained solid samples was carried out by X-ray fluorescence (XRF) using two methods. First method allows the quantification of all elements, including C, and it was conducted on a wavelength dispersive X-ray spectrometer, Rigaku ZSX Primus II, equipped with a Rh source, 4.0 kW power, and a Be window with a thickness of 30 microns. The XRF measurements were performed on pressed pellets of 10 mm thickness under vacuum, and data were analyzed using EZ-scan software combined with Rigaku’s SQX fundamental parameters software (standard less). The second method was used for high accuracy S content analysis, and the XRF analysis was performed on an EDAX Orbis Micro-XRF system equipped with a 30 mm^2^ silicon drift detector (SDD) that offers liquid nitrogen-free operation and high signal throughput with an energy resolution of 180 eV. The X-ray source consists of a Rh cathode operating at acceleration voltages between 10 and 35 kV. The samples were measured in low vacuum, around 10^–2^ mtorr, with an X-ray beam size of 2 mm in diameter, at 15 kV accelerating voltage, a current of 500 µA, and 60 s measurement time per spectrum. The samples were not pelletized, but measured as extracted from the reactor, having around 1 cm thickness. The attenuation length of carbon at S K edge (~ 2500 eV) is around 30 microns. Three random positions were averaged on each sample for a better statistic.

To determine the chemical composition of the liquid and solid samples, measurements were made by Inductively Coupled Plasma Optical Emission Spectroscopy (ICP-OES) using a Ciros and Arcos Vision ICP-OES equipment (Spectro, Germany) equipment. This instrument is equipped with a concentric nebulizer, a Scott spray chamber, a Rowland circle polychromator wavelength selector and a charge coupled device (CCD) detector. Before their analysis by ICP-OES, the samples were submitted to an acid digestion by using Anton Paar Multiwave 3000 (Austria) microwave digestor. For this, 50 mg of sample was digested in duplicate using 6 mL HNO_3_ (65%) and 1 mL HCl (37%). The program employed for the microwave digestion was the following: power increase to 900 W in 20 min and maintained at constant power for 25 min. After cooling, the sample digests were dissolved with Milli-Q water up to 50 mL. Finally, the sample was prepared for ICP-OES analysis by diluting them to 1:100 and 1:10 with HNO_3_ (1%). The quantitative analysis was carried out by preparing calibration curves for Zn (0.02–2 mg L^−1^, Bernd Kraft GmbH, Switzerland) and for S (0.5 mg L^−1^–50 mg L^−1^, Bernd Kraft GmbH, Switzerland). The ICP-OES operating conditions were the following: 1200 W RF power, radial plasma configuration, 0.8 L min^−1^ nebulizer flow rate, 15 L min^−1^ plasma gas flow rate, 30 s of reading time and 3 replicates for each reading. The selected wavelengths for the elements of interest were 180.731 nm (S) and 113.856 nm (Zn).

## Supplementary Information

Supplementary Information.

## Data Availability

The datasets generated and/or analyzed during the current study are available from the corresponding authors on reasonable request.
