# Supplementary material for: Carbon-supported ZnO materials for sulfur capturing in supercritical water
Source: Sci Rep. 2025 Apr 24;15:14239. doi: 10.1038/s41598-025-98741-2 (PMC12022147; doi:10.1038/s41598-025-98741-2)
Supplement: Supplementary file 1 — Supplementary Information. [file 41598_2025_98741_MOESM1_ESM.pdf]

# Carbon-supported ZnO materials for sulfur capturing in supercritical water

F. Maxim[1,*], G. S. Stoian[1], E. E. Toma[1], C. N. Borca[2], E. Muller[3],
I. Atkinson[4], L. Torrent[5,6], C. Ludwig[5,7], A. Testino[5,8,*]

[1] Institute of Physical Chemistry - Ilie Murgulescu, Laboratory of Chemical Thermodynamics, Splaiul Independentei 202, 060021 Bucharest, Romania;
[2] Paul Scherrer Institute, PSI Center for Photon Science, CPS-LSF, 5232 Villigen PSI, Switzerland;
[3] Paul Scherrer Institute, PSI Center for Life Sciences, LNB-EMF, 5232 Villigen PSI, Switzerland;
[4] Institute of Physical Chemistry - Ilie Murgulescu, Laboratory of Oxide Compounds and Materials Science, Splaiul Independentei 202, 060021 Bucharest, Romania;
[5] Paul Scherrer Institute, PSI Center for Energy and Environmental Sciences, CEE-LEP-CPM, 5232 Villigen PSI, Switzerland;
[6]University of Girona, Faculty of Sciences, Department of Chemistry, 17003 Girona, Spain;
[7]École Polytechnique Fédérale de Lausanne (EPFL), ENAC IIE GR-LUD, 1015 Lausanne, Switzerland ;
[8]École Polytechnique Fédérale de Lausanne (EPFL), STI-SMX, 1015 Lausanne, Switzerland

*Correspondence: fmaxim@icf.ro, andrea.testino@psi.ch

# Supplementary information

## Contents

Table S1. X-ray fluorescence (XRF) data of the investigated samples. .......... 2

Fig. S1. Fluorescence spectrum in the S $K_\alpha$ region, obtained via the first XRF method for the main samples of this study. .......... 2

Fig. S2. a) The picture of the C monolith used as support for impregnation showing how it is placed on the thermocouple protection tube to upload it in the reactor; b) The SEM micrograph of the support that displays the containing C fibers of the monolith; c) The XRD pattern of the C-monolith sample with graphite structure. .......... 3

Fig. S3. a) The on-line monitoring of the ratio between the conductivity measured at the outlet and inlet during the impregnation experiment with $Zn(NO_3)_2$; no substantial variations of the conductivity are registered during the time of the experiment; b) The on-line monitoring of the effluent conductivity variation during the sulfidation experiments with NaHS (aq) over ZnO/C sorbent material; the conductivity increases at the beginning of the experiment, stabilizes at a value with respect to the saturation of the adsorbent material, and then follows an increasing trend towards the end of the experiment. .......... 4

Table S1. X-ray fluorescence (XRF) data of the investigated samples.

| Sample | C, %[a] | S, %[a] | Zn, %[a] | Other, %[a] |
|---|---|---|---|---|
| **C** | 95.90 | 1.84 | - | 2.26 |
| **ZnO/C** | 94.47 | 1.74 | 0.19 | 3.60 |
| **C + NaHS** | 95.68 | 0.73 | - | 3.59 |
| **ZnO/C + NaHS** | 90.94 | 2.17 | 0.32 | 6.57 |

[a] mass percent from XRF.

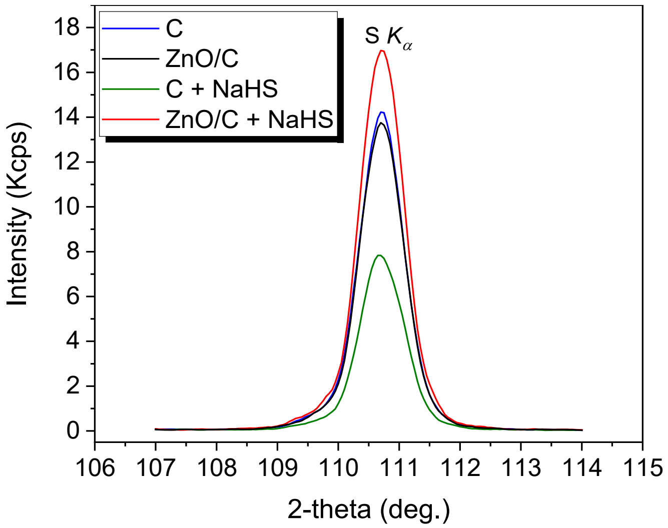


Fig. S1. Fluorescence spectrum in the S $K_\alpha$ region, obtained via the first XRF method for the main samples of this study.

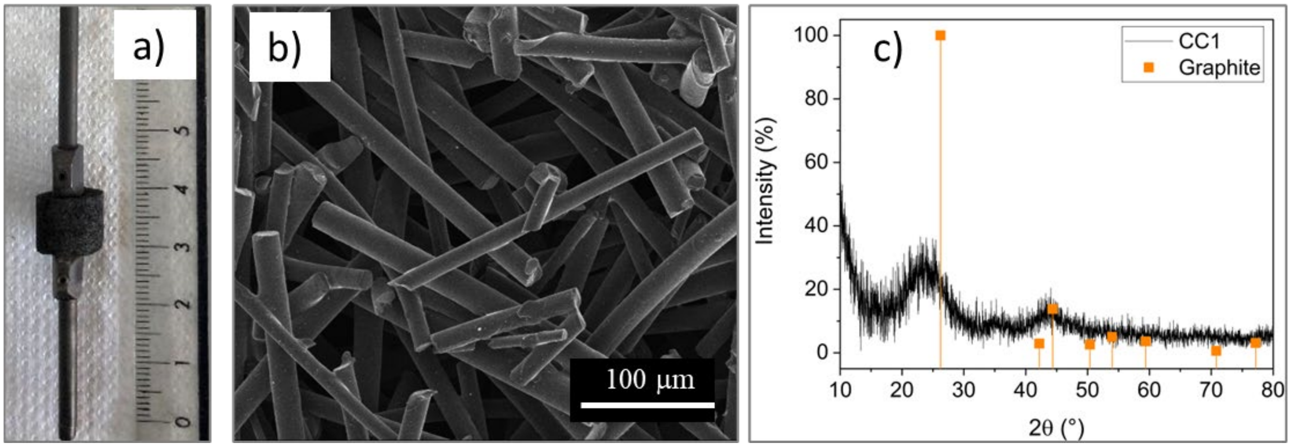


Fig. S2. a) The picture of the C monolith used as support for impregnation showing how it is placed on the thermocouple protection tube to upload it in the reactor; b) The SEM micrograph of the support that displays the containing C fibers of the monolith; c) The XRD pattern of the C-monolith sample with graphite structure.

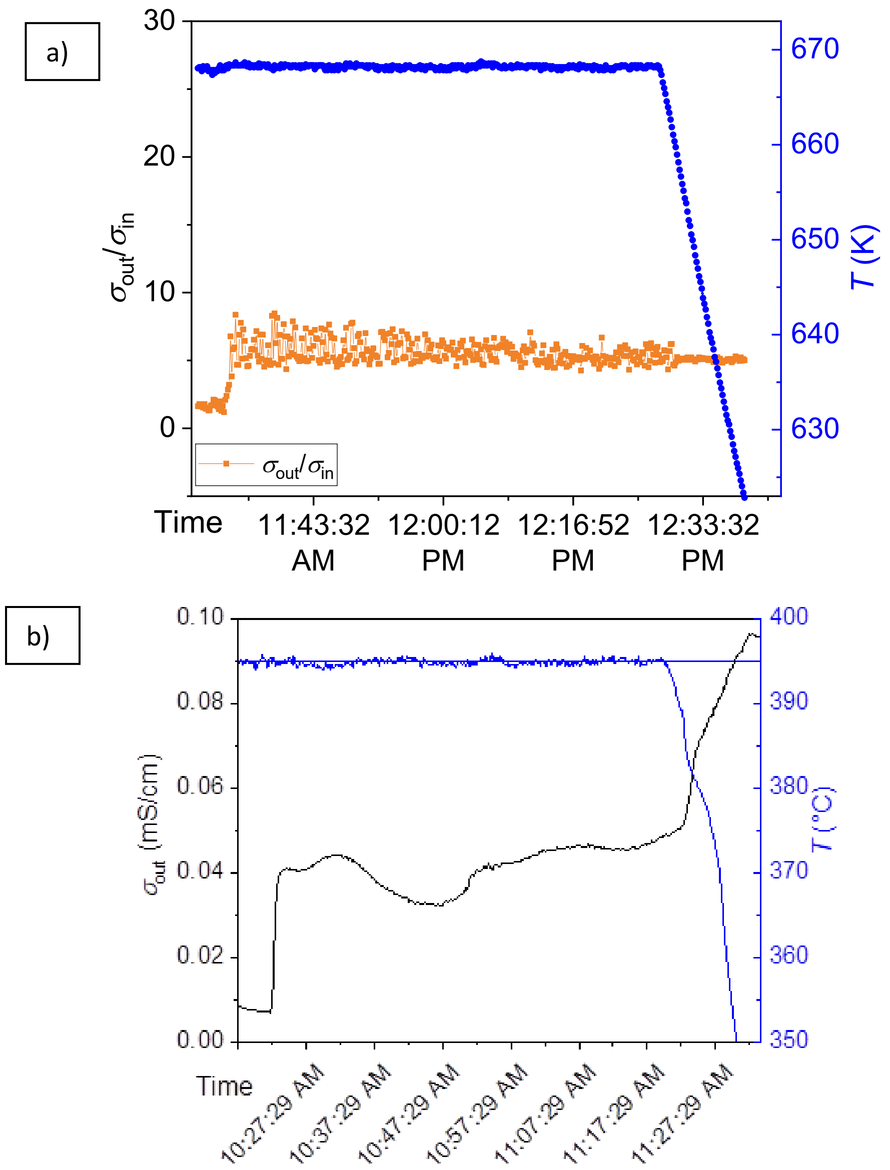


Fig. S3. a) The on-line monitoring of the ratio between the conductivity measured at the outlet and inlet during the impregnation experiment with $Zn(NO_3)_2$; no substantial variations of the conductivity are registered during the time of the experiment; b)The on-line monitoring of the effluent conductivity variation during the sulfidation experiments with NaHS (aq) over ZnO/C sorbent material; the conductivity increases at the beginning of the experiment, stabilizes at a value with respect to the saturation of the adsorbent material, and then follows an increasing trend towards the end of the experiment.
